# Development and Simulation-Based Validation of Biodegradable 3D-Printed Cog Threads for Pelvic Organ Prolapse Repair

**DOI:** 10.3390/ma18153638

**Published:** 2025-08-01

**Authors:** Ana Telma Silva, Nuno Miguel Ferreira, Henrique Leon Bastos, Maria Francisca Vaz, Joana Pinheiro Martins, Fábio Pinheiro, António Augusto Fernandes, Elisabete Silva

**Affiliations:** 1 LAETA, INEGI, Rua Dr. Roberto Frias s/n, 400, 4200-465 Porto, Portugal; tcsilva@inegi.up.pt (A.T.S.); nmferreira@inegi.up.pt (N.M.F.); fvaz@inegi.up.pt (M.F.V.); jamartins@inegi.up.pt (J.P.M.); fpinheiro@inegi.up.pt (F.P.); aaf@fe.up.pt (A.A.F.); 2 Faculty of Engineering, University of Porto, Rua Dr. Roberto Frias s/n, 400, 4200-465 Porto, Portugal; up202000174@edu.fe.up.pt; 3 School of Medicine and Biomedical Sciences, University of Porto, R. de Jorge de Viterbo Ferreira 228, 4050-313 Porto, Portugal

**Keywords:** pelvic organ prolapse (POP), biodegradable cog threads, finite element analysis (FEA), vaginal wall reinforcement

## Abstract

Pelvic organ prolapse (POP) is a prevalent condition, affecting women all over the world, and is commonly treated through surgical interventions that present limitations such as recurrence or complications associated with synthetic meshes. In this study, biodegradable poly(ϵ-caprolactone) (PCL) cog threads are proposed as a minimally invasive alternative for vaginal wall reinforcement. A custom cutting tool was developed to fabricate threads with varying barb angles (90°, 75°, 60°, and 45°), which were produced via Melt Electrowriting. Their mechanical behavior was assessed through uniaxial tensile tests and validated using finite element simulations. The results showed that barb orientation had minimal influence on tensile performance. In simulations of anterior vaginal wall deformation under cough pressure, all cog thread configurations significantly reduced displacement in the damaged tissue model, achieving values comparable to or even lower than those of healthy tissue. A ball burst simulation using an anatomically accurate model further demonstrated a 13% increase in reaction force with cog thread reinforcement. Despite fabrication limitations, this study supports the biomechanical potential of 3D-printed PCL cog threads for POP treatment, and lays the groundwork for future in vivo validation.

## 1. Introduction

Pelvic organ prolapse (POP) is a common pelvic condition characterized by the weakening of supporting muscles, tissues, and/or ligaments within the female pelvis. This loss of anatomical support leads to the descent of one or more pelvic organs (uterus, bowel, bladder, or vaginal vault) from their normal position, causing them to bulge into the vagina [1]. Nearly 50% of women experience some degree of POP [2], a condition with multifactorial causes resulting from the complex interaction of various key risk factors, like age, parity, genetic predisposition, obesity, operative vaginal delivery, chronic constipation, and heavy lifting [3].

Current POP treatment options include non-surgical approaches, such as pessaries, and surgical interventions. Surgical procedures can be performed via transvaginal, laparoscopic/robotic, or open approaches, using either native tissue repair or mesh augmentation. However, both strategies present notable limitations. Native tissue repairs are associated with high recurrence rates (16–29%) [4], while synthetic meshes have been linked to complications such as pain, erosion, and infection, with a 30% re-operation rate [5]. Long-term studies have shown significant mesh-related complications (MRCs), raising concerns about their safety and durability [6]. These issues ultimately led to the 2019 FDA ban on the use of synthetic mesh for transvaginal POP repair [7].

With a projected 48.1% increase in POP surgeries between 2010 and 2050, there is an urgent need for more effective and long-lasting repair strategies [8]. Among emerging alternatives, biodegradable cog threads have gained attention due to their minimally invasive application and favorable safety profile [9]. Originally developed for aesthetic procedures such as facial thread lifting, these threads enable tissue anchoring without general anesthesia and with reduced complication rates. Notably, cog threads have been used in gynecology since 2008 for tissue approximation in laparoscopic procedures such as myomectomies and hysterectomies [10]. This background supports their potential for POP treatment, offering a less invasive and bioresorbable solution to reinforce weakened pelvic tissues—specifically, the vaginal canal [11].

Recent research has highlighted the promising application of biodegradable cog threads made from poly(ϵ-caprolactone) (PCL) for reinforcing the vaginal wall in the treatment of POP. PCL has gained particular attention among available thread materials due to its high flexibility, biocompatibility, and slow degradation rate. Compared to polydioxanone (PDO) and poly-L-lactic acid (PLLA) threads, PCL is associated with less patient discomfort and a longer resorption period of approximately 12 to 18 months. Its established biodegradability and FDA approval for various medical devices further support its clinical safety [12].

In addition to its favorable material properties, several studies have reported promising biological and mechanical outcomes. Ex vivo investigations demonstrated a significant increase in tissue strength following cog thread implantation [11], while finite element analyses confirmed the mechanical viability of the technique [13]. Furthermore, an in vivo study using ovine models reported an approximate 42% increase in intravaginal tensile strength after cog thread placement [14], underscoring their potential to enhance pelvic support through minimally invasive means.

The main objective of this study was to develop and validate biodegradable 3D-printed cog threads for POP repair. This included the design and manufacture of the threads using Melt Electrowriting (MEW) with PCL, supported by the development of a custom cutting tool to achieve precise barb geometries. Mechanical testing and finite element simulations were then used to evaluate their reinforcement potential in pelvic anatomical models.

To this end, the study was structured into successive stages, combining experimental characterization and computational simulations; the mechanical behavior of the printed cog threads was evaluated through uniaxial testing, and the resulting data were used to validate numerical simulations of cog threads with different cutting angles (90°, 75°, 60°, and 45°) under tensile loading. Additionally, the biomechanical response of the vaginal canal and uterus was simulated under cough pressure conditions, considering both healthy and damaged tissues, with and without reinforcement using cog threads of different cutting angles, and the resulting antero-posterior displacement of the anterior vaginal wall was analyzed. Finally, a ball burst test was simulated in the vaginal canal to evaluate and compare tissue resistance in the presence and absence of cog thread reinforcement, with reaction forces analyzed to assess the mechanical contribution of the threads in each scenario.

## 2. Materials and Methods

### 2.1. Commercial Cog Threads

**Figure 1:**
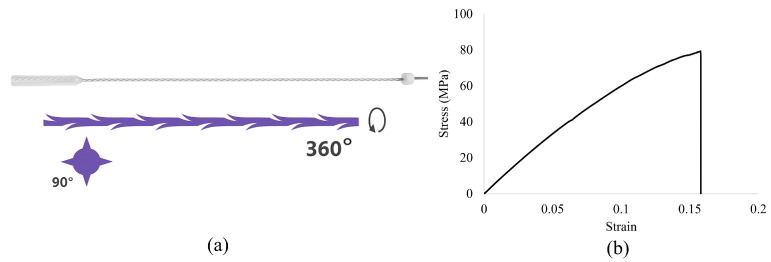
(**a**) Commercial cog thread model 4D-1W-18 G R (cannula); (**b**) Representative stress–strain curve of commercial cog thread, highlighting its mechanical response under uniaxial tensile loading (adapted from [13]).

Commercially available 360° 4D barb threads (PCL-19G-100) (Figure 1a), made of PCL and supplied by Yastrid (Shanghai, China), served as the basis for the design and development of the threads utilized in this study. These commercial threads are typically supplied in sterile packs, each containing two threads pre-loaded into an L-type cannula and a 19G stainless steel needle. Once implanted, their expected duration is between 2 and 3 years [11].

Each commercial thread measures 160 mm in length and has a diameter of 630 µm. Their design features four cutting directions, evenly spaced at 90° intervals and separated by 1600 µm. A 45° cog cut angle is another critical feature, optimizing the thread’s ability to support and reinforce vaginal tissue. The cog thread’s cutting depth is consistently maintained at 200 µm [13]. Figure 1b illustrates the stress–strain response of the commercial cog thread, reaching a peak stress close to 85 MPa before failure [13].

### 2.2. Cog Thread Cutting Tool

The cutting tool was designed using SolidWorks 2023 (Dassault Systèmes, Vélizy-Villacoublay, France), a Computer-Aided Design (CAD) software. Its components were manufactured using Fused Deposition Modeling (FDM) 3D printing technology, specifically with the Prusa Mk3S and Elegoo Neptune 4 Pro printers (Elegoo Inc., Shenzhen, China). Notably, the tool’s components were printed using Polylactic Acid (PLA) material.

**Figure 2:**
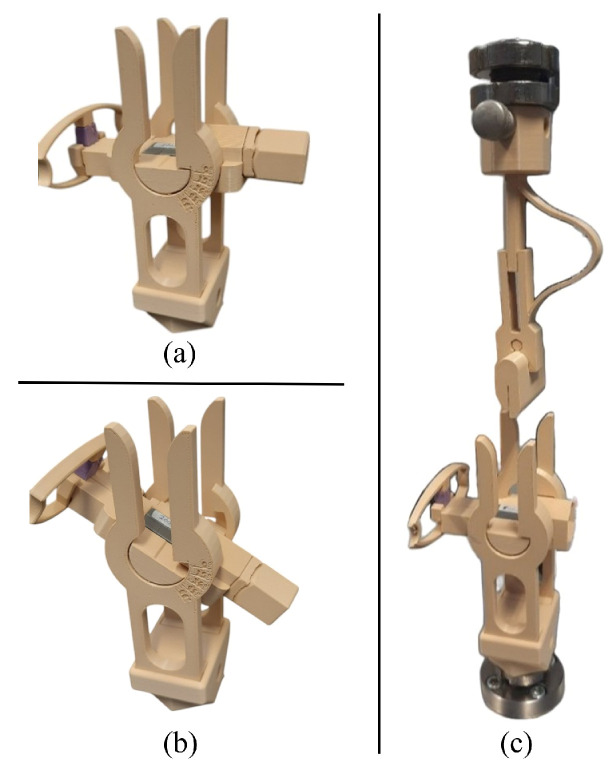
(**a**) Cutting tool positioned at a 90° angle; (**b**) cutting tool positioned at a 60° angle; (**c**) complete tool used for making cuts in the threads.

The tool, shown in Figure 2, was developed to accurately position the wire and control the cutting angle. In this design, the wire support remains fixed, while the blade adjusts to the required angle. A ramp-shaped guide path directs the blade into the correct orientation immediately before cutting and returns it to its original position afterward. A custom-designed spring applies the necessary cutting force while allowing the blade to move along the guide. The ramp not only ensures proper blade alignment, but also defines the final cutting angle at the moment of operation.

### 2.3. PCL Threads and Printing Machine

PCL filaments were printed using a custom-built MEW system developed under the SPINMESH project (FCT-funded). The device features a modular design with an XY-moving collector, a Z-moving print head, and a high-voltage generator (60 kV–150 W). A key modification included the addition of a heated print bed, which enhanced interlayer adhesion by maintaining the filaments in a semi-molten state upon deposition. This adjustment improved printing accuracy and structural integrity, reducing the risk of filament detachment and resulting in more uniform and mechanically robust scaffolds [13]. The printing process was consistently carried out in 200 mm sections.

The polymer selected for this study was Facilan™ PCL filament (3D4Makers), chosen for its FDA approval and proven suitability for 3D printing. It has a density of 1.1 g/cm^3^ and an initial diameter of 1.75 mm. As a semi-crystalline material, it presents a low melting point and fast solidification—features that support precise extrusion. Its mechanical properties (tensile strength of 45 MPa and modulus of 350 MPa) make it suitable for soft tissue-related biomedical applications [15].

The optimized printing parameters for the extrusion of PCL filaments are detailed in Table 1. The average diameter of the resulting printed PCL filament was measured at 598.33 µm.

**Figure 3:**
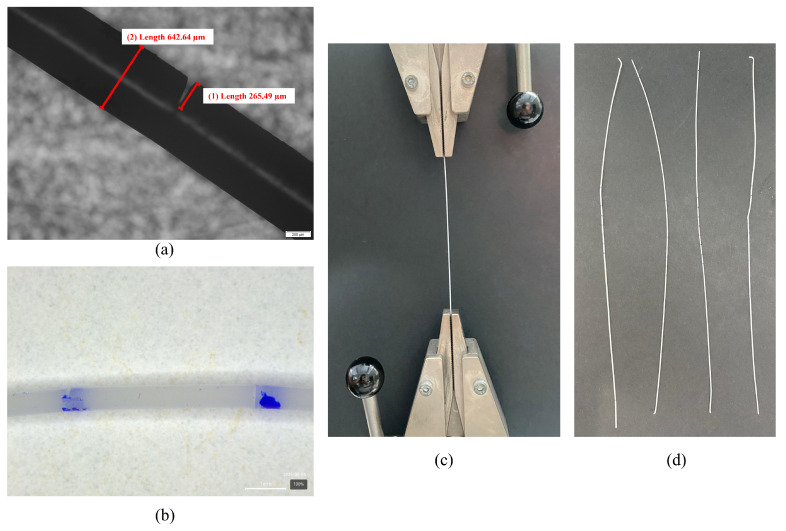
(**a**) Microscopic analysis of the cut; (**b**) a magnified view showing two 90° cuts on the thread, made at distinct positions and orientations; (**c**) the experimental setup for the uniaxial mechanical characterization of cog threads; (**d**) representative examples of the cog thread types evaluated in the uniaxial tensile tests.

Each wire sample was cut in four different radial directions, spaced 90° apart, in order to replicate the geometry of commercially available 360° 4D barb threads (PCL-19G-100). The cutting process was performed using a Mecmesin Multitest 2.5-dV universal testing machine (Mecmesin, Slinfold, UK) equipped with a 100 N Mecmesin AFG force gauge. The developed cutting tool was mounted on the testing apparatus, and cuts were made by manually advancing the crosshead until the desired depth was reached. Wire samples were cut at predefined angles of 90°, 75°, 60°, and 45°. For each cutting angle, three wire samples were prepared and analyzed, and the final values presented in the results correspond to the mean of those three samples. The resulting cuts were subsequently analyzed via microscopy, as illustrated in Figure 3a,b.

### 2.4. Uniaxial Tensile Testing of Cog Threads

To characterize the mechanical properties of the PCL cog threads, uniaxial tensile tests were performed. Figure 3c illustrates the experimental setup employed for these tests. This setup enabled precise measurement of load, crucial for determining the Young’s modulus and strain behavior of the PCL threads. Each thread sample was carefully mounted in the testing machine’s grips, ensuring axial alignment for the application of uniaxial tensile loads. A consistent crosshead speed of 10 mm/min was maintained throughout all tests to ensure data comparability. Load and displacement data were synchronously recorded by the integrated data acquisition system. Representative examples of the cog thread types evaluated are shown in Figure 3d.

### 2.5. Computational Models

#### 2.5.1. Cog Thread Computational Model

**Figure 4:**
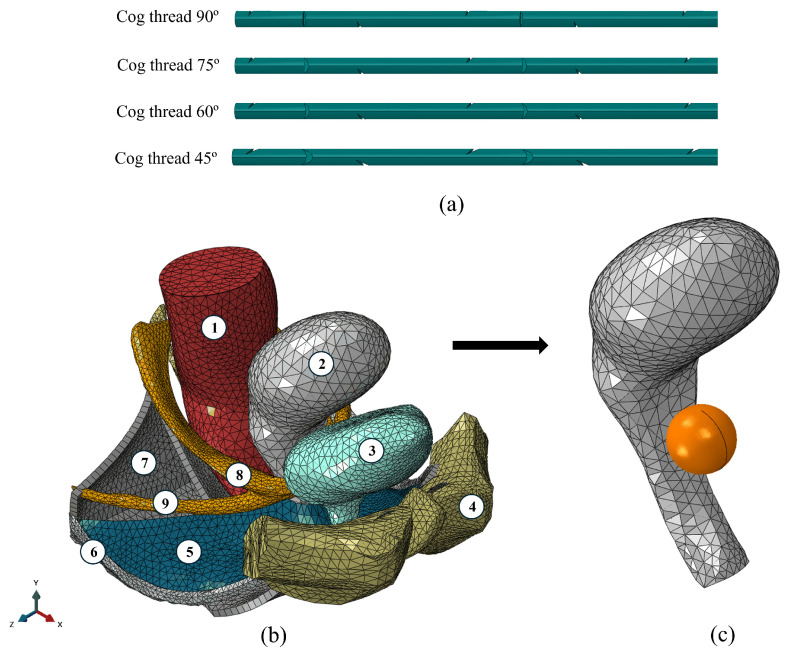
(**a**) The developed computational models of cog threads; (**b**) a 3D computational model of the pelvic cavity of an asymptomatic woman. (1) rectum; (2) uterus; (3) bladder; (4) symphysis pubis; (5) pelvic fascia; (6) arcus tendineous fasciae pelvis; (7) LAM; (8) USLs; (9) CLs [16]. (**c**) A simplified computational model of the pelvic cavity considered in this study.

In this study, models were initially developed using SolidWorks software v2023 and were subsequently imported into Abaqus software v2025 for further associated testing. Threads with varying cutting angles were each generated with four cutting directions, positioned at 90° intervals and separated by 1600 µm. Additionally, the cog thread’s cutting depth was consistently maintained at 200 µm. These models aimed to facilitate a comparison with experimental results, thereby facilitating investigation of whether the cutting geometry influences vaginal canal reinforcement. All threads were created from the same 80 mm filament with a 600 µm diameter. The C3D10 element type was utilized for the cog threads in Abaqus. The developed models are represented in Figure 4a.

The constitutive law of a material defines how stress (σ) and strain (ε) are related. For linear elastic materials, this relationship is determined by the Young’s modulus and Poisson’s ratio, and is mathematically represented by Equation (1):

(1) $$\begin{array}{c} \sigma =E\epsilon \end{array}$$

where *E* quantifies the material’s stiffness under elastic deformation [17].

The Young’s modulus measures a material’s resistance to elastic deformation, and corresponds to the initial slope of the stress-strain curve. In this study, simulations focused only on the elastic range to prevent permanent deformation [18]. This approach ensures that a linear elastic constitutive model is appropriate and accurately represents the material behavior under the investigated conditions, where plastic deformation is explicitly avoided.

The material properties for the biodegradable cog threads were defined in Abaqus^®^, incorporating a Young’s modulus of 367.69 MPa (determined from the experimental stress-strain curve of the uncut filament) and a Poisson’s ratio of 0.3.

#### 2.5.2. Pelvic Cavity Computational Model

The model was developed by Brandão et al. [19] based on data from a 24-year-old nulliparous woman. It includes several important anatomical structures, such as the bladder, uterus, rectum, levator ani muscles (LAMs), pelvic fascia, tendon arch, uterosacral ligaments (USLs), and cardinal ligaments (CLs), as shown in Figure 4b. The Research Ethics Committee at Centro Hospitalar de São João-EPE, Porto, Portugal (protocol: IRB138/19) granted full approval for this study. This 3D model was validated when it was used to simulate sacrocolpopexy with synthetic implants [20] and to estimate the in vivo biomechanical properties of the continent and incontinent woman’s bladder [21]. Figure 4c presents the simplified model utilized during the study. This particular model consists solely of the uterus and a rigid sphere, which was employed to replicate the conditions of a ball burst test. An additional test was conducted with the simplified model without the sphere, applying a cough pressure of 160 cmH_2_O, selected to simulate a worst-case scenario, as coughing generates some of the highest physiologically relevant intra-abdominal pressures [22].

In order to approximate the mechanical constraints that the uterus is generally subjected to by surrounding anatomical structures, boundary conditions were applied in the simplified computational model, as shown in Figure 4c. A fixed support (encastre) was imposed at the lower part of the vaginal canal to simulate pelvic attachment. Additionally, displacement was restricted in the x (antero-posterior) and z (lateral) directions in the posterior region of the model to mimic the mechanical constraint exerted by the rectum. A uniform pressure load was applied to the anterior uterine wall to represent bladder-induced pressure. Finally, a frictionless constraint was defined between the uterus and the cog threads to ensure full contact without relative motion. In the ball burst test simulation, a rigid analytical sphere was positioned above the anterior vaginal wall and displaced in the supero-inferior direction. The vaginal canal was fixed at its base, and contact between the sphere and the tissue was modeled as frictionless. The sphere was defined as undeformable to simplify loading application and ensure stable computation.

Hyperelastic materials, which exhibit significant elasticity and can undergo large deformations, are described using strain energy potential [23]. Pelvic floor tissues fall into this category, showing a nonlinear stress–strain relationship where stress rises sharply at high strain levels until the material reaches its failure strength, potentially affecting its function [24].

To model the mechanical behavior of the uterus and vaginal canal, hyperelastic constitutive models like Ogden are frequently used. These models are designed for incompressible materials and effectively approximate tensile behaviour, making them particularly valuable for studying biomechanical responses [16]. While these models do not incorporate effects like viscosity, temperature, or time-dependency, they still provide reliable predictions for analyzing the mechanical properties of pelvic cavity tissues [25].

The Ogden constitutive model is characterized by Equation (2) [26]:

(2) $$\begin{array}{c} W=\sum_{i=1}^{n}\mu_{n}\frac{\lambda_{1}^{\alpha_{n}}+\lambda_{2}^{\alpha_{n}}+\lambda_{3}^{\alpha_{n}}-3}{\alpha_{n}} \end{array}$$

This model is highly adaptable and proficient in describing the deformation behaviour of soft tissues. Material parameters such as $\mu_{n}$ and $\alpha_{n}$ are obtained through experimental data, ensuring accurate simulations of pelvic biomechanics. Additionally, *n* represents the number of material parameters [26]. The material parameters for the pelvic structures were derived from experimental studies on cadavers without pelvic dysfunction. These parameters, detailed in Table 2, enabled precise modeling of the stress–strain response within the pelvic cavity under various conditions [20,21].

**Figure 5:**
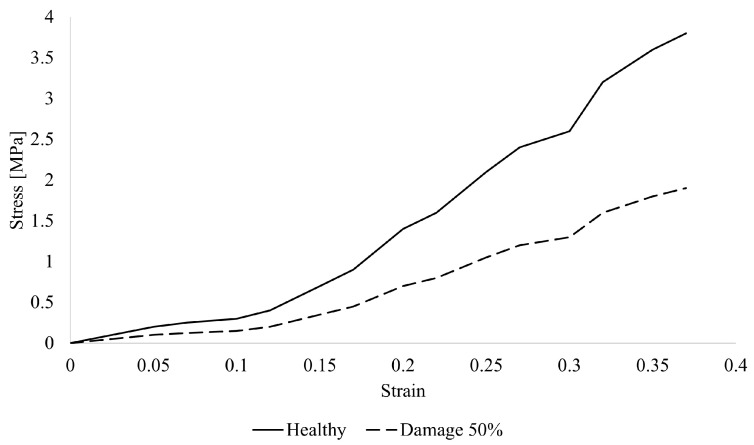
Stress–strain curves for healthy and 50% damaged anterior vaginal wall.

In this study, simulations were conducted with a 50% reduction in the stiffness of the anterior vaginal wall (Figure 5), representing a damaged state relative to the healthy model. This 50% damage effectively indicates a proportional decrease in the material’s resistance.

#### 2.5.3. Ball Burst Test

Building on previous studies that employed the ball burst test to demonstrate the reinforcing effect of cog threads on vaginal tissue, a similar mechanical assessment was replicated using a more anatomically realistic setup, as shown in Figure 4c. Instead of isolated tissue samples, the entire vaginal canal was utilized to capture the global mechanical response. A rigid analytical sphere (radius = 10 mm) was used to apply controlled displacement to the anterior wall of the vaginal canal, simulating conditions similar to the ball burst test, but within the anatomical context. This approach allowed us to evaluate whether the insertion of cog threads effectively reinforces the vaginal wall under loading conditions, as previously suggested in simplified experimental models.

## 3. Results

### 3.1. Uniaxial Tensile Testing of Cog Threads

**Figure 6:**
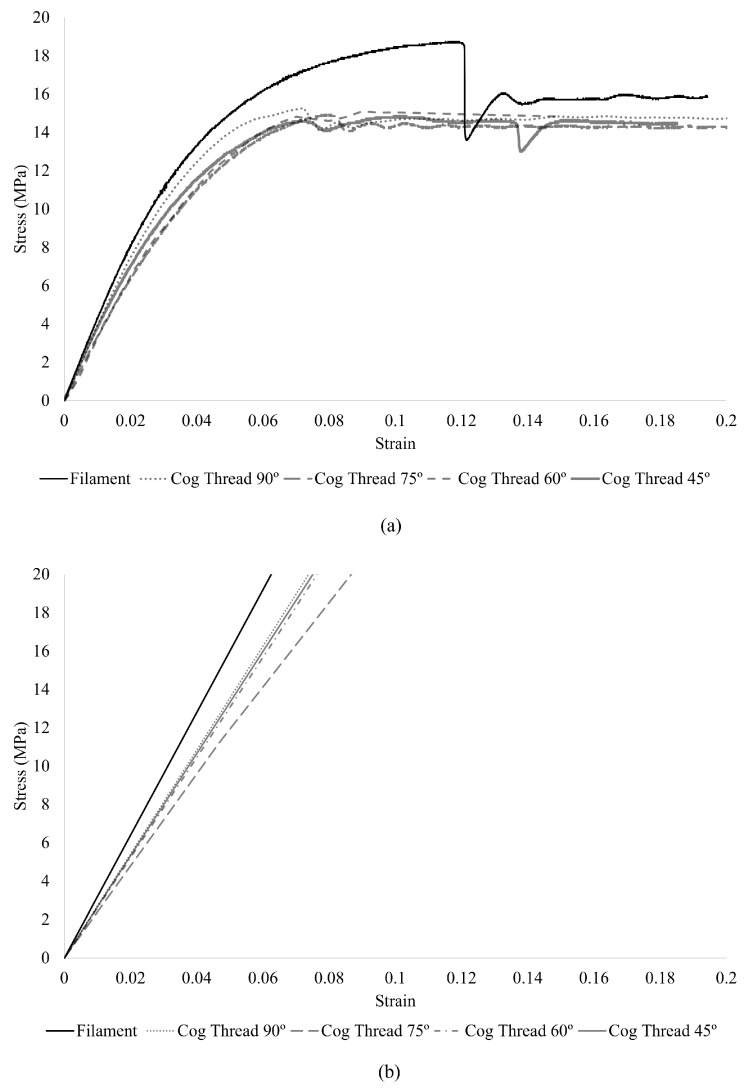
Uniaxial tensile test results of cog threads: (**a**) experimental stress–strain curves; (**b**) numerical stress–strain curves.

The stress–strain curves obtained from uniaxial tensile tests are presented in Figure 6a. These tests were conducted using a Mecmesin Multitest system, equipped with an ILC 100N load cell and a consistent crosshead speed of 10 mm/min.

The uniaxial tensile tests showed that the uncut filament exhibited higher mechanical resistance compared to filaments with various cutting angles. While a clear correlation between cutting angle and mechanical resistance was not established, the 90° angle showed the highest resistance, whereas the 75° angle resulted in the lowest stiffness. However, the overall differences in mechanical properties among the various cutting angles were not substantial.

Figure 6b displays the stress–strain results derived from numerical simulations of the developed computational cog thread models, enabling a direct comparison with the experimentally obtained data.

### 3.2. Numerical Simulation of Anterior Vaginal Wall

To evaluate the mechanical stability provided by different reinforcement strategies, a cough pressure of 160 cmH_2_O was applied to the anterior wall of the vaginal canal, and the resulting displacement was measured [22]. The Table 3 presents the displacement values for a healthy model, a damaged model without reinforcement, and four reinforced models using cog threads inserted at different angles (90°, 75°, 60°, and 45°). All reinforcement configurations consistently reduced antero-posterior displacement compared to the unreinforced damaged model, with values approaching those of healthy tissue. Among the cog thread configurations, the variation in angle had minimal influence, with displacements ranging narrowly between 7.071 mm and 7.078 mm.

**Figure 7:**
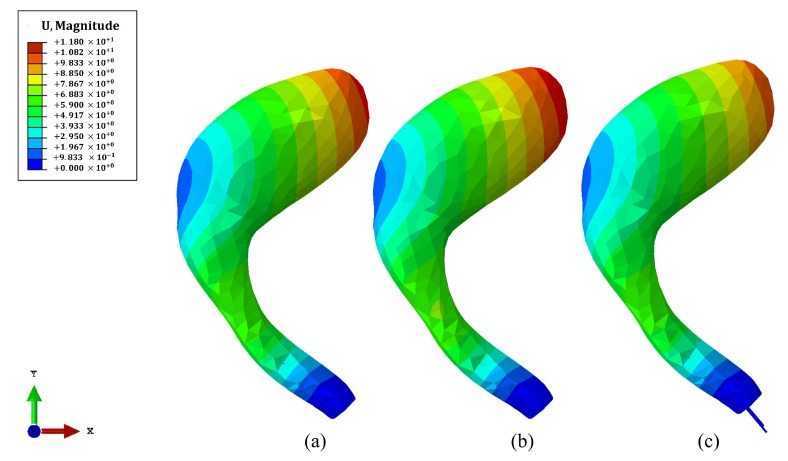
The magnitude of displacement (in mm) of the anterior vaginal wall under a pressure of 160 cmH_2_O is shown for (**a**) the healthy model without cog threads, (**b**) the damaged model without cog threads, and (**c**) the damaged model reinforced with cog threads with a 45° cutting angle. Warmer colors indicate higher displacement, while cooler colors correspond to lower displacement.

Figure 7 displays the computational results for the displacement of the anterior vaginal wall under a pressure of 160 cmH_2_O, comparing various structural conditions.

Figure 7a illustrates the behavior of the healthy model without cog threads, which serves as the reference for normal physiological displacement.

Figure 7b represents the damaged model without any reinforcement, revealing an increase in displacement attributed to tissue weakening. In contrast, Figure 7c depicts the impact of cog thread reinforcement with a 45° cutting angle in the damaged model. The introduction of cog threads reduces deformation compared to the unreinforced scenario, bringing the displacement pattern closer to that of the healthy model and demonstrating the mechanical advantages of reinforcement.

### 3.3. Bull Burst Test

**Figure 8:**
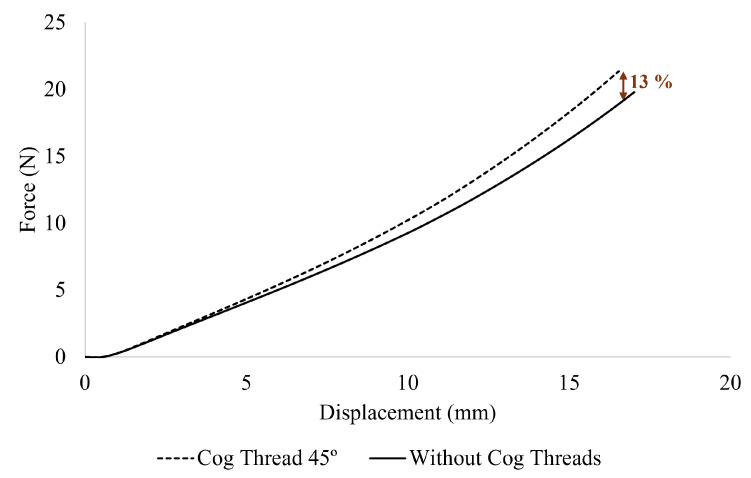
Force–displacement curves obtained for simulations with and without cog thread reinforcement. Solid line: vaginal wall reinforced with cog threads; dashed line: without reinforcement.

The mechanical response of the vaginal wall was evaluated through numerical simulations by imposing a displacement using a rigid analytical sphere. The aim was to assess the effect of reinforcement with cog threads on tissue resistance. As shown in Figure 8, the force–displacement curves indicate a higher reaction force when cog threads are present, compared to the unreinforced case. Notably, a 13% increase in reaction force was observed at the end of the displacement for the reinforced model, suggesting enhanced mechanical resistance to deformation. These results are consistent with experimental findings previously reported in the literature [13].

## 4. Discussion

Given the limitations and complications associated with synthetic meshes, which typically require surgical implantation to treat advanced stages of POP, cog threads offer a promising, minimally invasive alternative with favorable biocompatibility and mechanical potential. Unlike conventional approaches that aim to reconstruct major support structures, cog threads are intended for local reinforcement of the vaginal wall in early-stage prolapse, potentially delaying or avoiding more invasive interventions. The primary aim of this work was to explore and validate a novel reinforcement strategy for the vaginal canal using biodegradable cog threads, specifically designed to address the structural weakening associated with POP.

The uniaxial tensile tests of the printed PCL cog threads revealed that although cutting geometry affects mechanical strength, the differences between angles (90°, 75°, 60°, and 45°) were relatively small. This suggests that, under pure tensile loading, the orientation of the barbs has a limited influence on the mechanical resistance of the cog thread. The cog thread cut at 90° exhibited the highest stiffness, while the 75° cut showed the lowest, yet the overall variability was modest. These results, although relevant for understanding the isolated behavior of the threads, may not fully translate to performance in situ.

When comparing the cog threads developed in this study to commercially available ones, it was evident that, although both were made from PCL, the mechanical performance of the 3D-printed threads produced in our laboratory was significantly lower [13]. This discrepancy likely results from differences in manufacturing methods, processing parameters, and microstructural characteristics. Commercial cog threads are produced under tightly controlled industrial conditions that ensure precise geometry, optimized crystallinity, and enhanced mechanical properties. In contrast, the MEW-printed threads manufactured in our lab showed reduced stiffness and tensile strength, which may have limited their reinforcement capacity.

Indeed, when simulating the anatomical scenario of the vaginal canal under a pressure of 160 cmH_2_O (representative of cough pressure conditions) [22], a more distinct effect of cog thread reinforcement was observed. All configurations with cog threads led to a reduction in anterior wall displacement compared to the damaged model without reinforcement. Interestingly, the displacements in the reinforced models were not only lower than those in the unreinforced damaged condition, but even slightly below the values of the healthy model. This indicates that the cog threads may effectively restore, or even exceed, native tissue resistance under pressure. Moreover, the cutting angle had a negligible influence in this context, suggesting that once the threads are embedded in tissue, the overall mechanical benefit becomes dominated by the presence of the anchoring barbs, rather than their exact angle.

The ball burst simulation, implemented here to replicate prior experimental work in a more anatomical setting, further confirmed the reinforcing role of the cog threads. In our model, the presence of cog threads led to a 13% increase in reaction force, particularly at higher displacements, where the threads become mechanically engaged. At lower displacements, the tissue absorbs most of the deformation, and the contribution of the threads is minimal. This nonlinearity reflects the progressive load-sharing behavior between the threads and the surrounding tissue. These findings align with previous ex vivo studies [11,13], which demonstrated that these threads reduce the vaginal wall’s deformability under load. Additionally, studies using the ball burst test reported a reinforcement effect of about 26% with cog thread insertion, highlighting their mechanical contribution. In our study, under similar intent but with a more anatomically realistic model, we observed only a 13% increase in reaction force. Several factors may explain this difference. Earlier tests used small tissue samples with cog threads inserted perpendicularly to the loading direction, directly engaging the threads from the start of deformation. In our setup, a rigid sphere applied displacement against the entire vaginal canal, distributing the mechanical load over a larger tissue area, which made the reinforcement effect of the threads less immediately apparent. Therefore, both geometric and boundary condition differences must be considered when interpreting and comparing these outcomes. It is important to note that vaginal tissue exhibits inherently anisotropic and hyperelastic mechanical behavior. While our current modeling approach employs a hyperelastic material model suited for capturing the large deformations that are typical of soft tissues, anisotropy was not considered. Incorporating an anisotropic hyperelastic model could better replicate the complex biomechanical response by accounting for directional dependencies in tissue strength and deformation capabilities.

Despite the promising findings, this study has several limitations. Experimentally, the small number of samples (*n* = 3) and the variability introduced during the manual cutting of the cog threads limit reproducibility and prevent statistical analysis. Furthermore, the mechanical characterization in this study was restricted to uniaxial tensile testing, which does not fully represent the complex loading conditions in the pelvic floor, such as shear, cyclic, and fatigue loading.

Computationally, simplifications were made regarding contact interactions (frictionless and no slippage), and the threads were modeled as linear elastic, although PCL is known to exhibit viscoelastic behavior. Another limitation lies in the finite element model, which was based on anatomical data from a nulliparous woman, and may not fully represent the pelvic anatomy of the typical POP population. Moreover, biological integration processes such as inflammation, fibrosis, and tissue remodeling were not included. Finally, the observed 13% increase in reaction force should be interpreted with caution, as it may lie near the threshold of numerical sensitivity.

Overall, the findings confirm the feasibility of using MEW-printed cog threads for mechanical reinforcement of prolapsed tissue. The negligible influence of barb angle provides flexibility in design, and the successful integration of anatomical simulations opens a pathway toward more clinically relevant testing.

In future work, further refinement of the fabrication process and the inclusion of in vivo validation would be crucial to support clinical translation. Moreover, assessing the degradation behavior of the threads through dedicated degradation tests over time and their integration with surrounding tissues will be essential to fully characterize their long-term efficacy and safety.

## Figures and Tables

**Table 1:** Printing parameters of PCL filaments.

Printing Bed Voltage (kV)	Nozzle-to-Bed Distance (mm)	Nozzle Diameter (µm)	Extrusion Speed (mm/min)
4.2	1	600	1000

**Table 2:** Material parameters for different pelvic structures (adapted from [20]).

Structures	$\alpha_{k}$	$\mu_{k}$	Model
Vagina and Uterus	$\alpha_{1}=-3.41$	$\mu_{1}=-92.24$	Ogden (N = 3)
	$\alpha_{2}=-0.66$	$\mu_{2}=39.29$	
	$\alpha_{3}=-6.48$	$\mu_{3}=54.68$	

**Table 3:** The antero-posterior displacement (in mm) of the anterior vaginal wall under a pressure of 160 cmH_2_O was assessed for various reinforcement conditions. The results are presented for three scenarios: a healthy model, a damaged model without reinforcement, and a damaged model reinforced with cog threads at different cutting angles (90°, 75°, 60°, and 45°).

Antero-Posterior Displacement of the Anterior Vaginal Wall (mm)					
**Healthy**	**Vaginal Canal with Damage**				
	**Without Cog**	**Cog Thread 90°**	**Cog Thread 75°**	**Cog Thread 60°**	**Cog Thread 45°**
7.489	7.857	7.078	7.072	7.073	7.071

## Data Availability

The original contributions presented in this study are included in the article. Further inquiries can be directed to the corresponding author.

## Abbreviations

The following abbreviations are used in this manuscript:

CAD	Computer-Aided Design
CLs	Cardinal ligaments
FDA	Food and Drug Administration
LAMs	Levator ani muscles
MEW	Melt Electrowriting
MRCs	Mesh-related complications
PCL	Polycaprolactone
PDO	Polydioxanone
PLA	Polylactic acid
PLLA	Poly-L-lactic acid
POP	Pelvic organ prolapse
USLs	Uterosacral ligaments
ε	Strain
σ	Stress
